# A203 A CASE OF PEDIATRIC CROHN’S DISEASE AND ADENOMATOUS POLYPOSIS AT DIAGNOSIS: A COLLISION OF PHENOTYPES

**DOI:** 10.1093/jcag/gwae059.203

**Published:** 2025-02-10

**Authors:** A Gopan, I Siddiqui, E I Benchimol

**Affiliations:** Gastroenterology, Hepatology and Clinical Nutrition, The Hospital for Sick Children, Toronto, ON, Canada; Gastroenterology, Hepatology and Clinical Nutrition, The Hospital for Sick Children, Toronto, ON, Canada; Gastroenterology, Hepatology and Clinical Nutrition, The Hospital for Sick Children, Toronto, ON, Canada

## Abstract

**Background:**

The co-occurrence of inflammatory bowel disease (IBD) and adenomatous polyposis syndrome is uncommon.

**Aims:**

To highlight the challenges of diagnosis and management of a rare case of concurrent adenomatous polyps in a child with newly diagnosed Crohn’s disease.

**Methods:**

Review of patient charts, endoscopic and histopathology images from electronic medical records.

**Results:**

An 11-year-old boy of Korean origin presented with a 6-month history of periumbilical abdominal pain, diarrhea, 5 kg weight loss, and a perianal abscess, which spontaneously ruptured the week prior. Family history revealed a father with uninvestigated vague abdominal symptoms and increased stool frequency for a few years. His mother had two adenomatous colonic polyps removed in the preceding year at age 43. There was no family history of IBD, polyposis syndrome or early colorectal cancer.

Physical examination revealed weight-for-age Z-score of -1.55 and height Z-score of 0.53 (with normal growth velocity), mild right lower quadrant abdominal tenderness and a healing perianal abscess.

Colonoscopy revealed nodularity from sigmoid colon to cecum, with a few deep ulcerations in the sigmoid, transverse and ascending colon. Additionally, there were prominent polyps in the transverse and ascending colon, with 3-4 sessile large polyps (>1cm) and numerous smaller polyps scattered between the sigmoid to cecum ***(figures 1A & B)***. Ileocecal valve was bulky, and ileoscopy revealed deep ulcers and nodularity. Histopathology revealed multiple non-necrotizing epithelioid granulomas without enterocolitis in duodenum and colon. Surprisingly, foci of bi- and tri-crypt superficial adenomas were noted in colonic biopsies ***(figures 1C & D).*** Magnetic resonance enterography demonstrated extensive active inflammation in the mid and distal ileum, narrowing in the ileocecal region, and an inter-sphincteric perianal abscess.

We commenced monotherapy with subcutaneous adalimumab using standard induction and maintenance dosing. He has been referred to a specialized polyposis syndrome clinic for genetic testing (results pending) and ongoing cancer surveillance.

**Conclusions:**

Adenomatous polyps are extremely rare at diagnosis in pediatric IBD and most polyps in this setting are presumed to be inflammatory pseudo-polyps. Their presence raises suspicion of adenomatous polyposis syndrome. Adequate biopsies are crucial for accurate diagnosis and for guiding surveillance and treatment. A detailed family history of colorectal disease is essential. Vitale V et al (2016) have described a hamartomatous polyp with IBD at diagnosis in a 6y old. This is to the best of our knowledge the first reported case of pediatric IBD with colonic adenomatous polyposis at diagnosis. Anti-TNF medications are presumed safe in this setting and genetic evaluation can provide insights into syndromic associations.

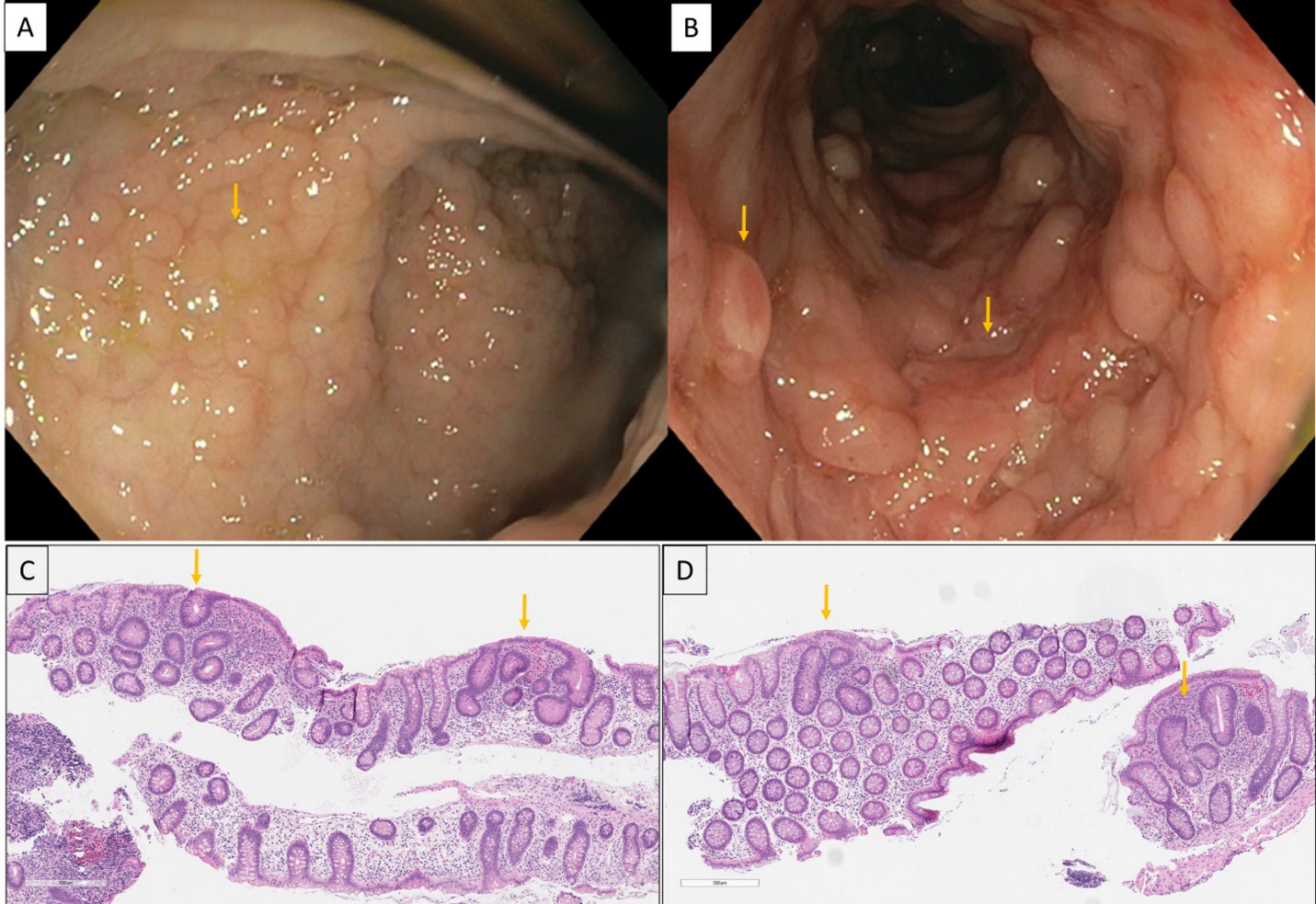

Figure 1: A) Nodularity in sigmoid colon which possibly represents nascent polyps; B) Larger polypoidal structures interspersed between pseudo polyps and ulceration in ascending colon; C) & D) Hematoxylin and eosin stain showing biopsies of the cecum and sigmoid colon (respectively), with foci of low-grade dysplasia involving 2-4 crypts (arrows), representing superficial tiny adenomas. Of note is the surrounding unremarkable mucosa without features of chronic or active Crohn’s disease [4x magnification, both images].

**Funding Agencies:**

None

